# Adherence to dental home care in dogs with periodontitis: a post-treatment survey

**DOI:** 10.1186/s13028-023-00718-6

**Published:** 2023-12-19

**Authors:** John Svärd, Karolina Brunius Enlund

**Affiliations:** Department of Clinical Sciences, Faculty of Veterinary Medicine and Animal Science, Swedish University of Agricultural Sciences, Uppsala, Sweden

**Keywords:** Compliance, Periodontal disease, Tooth brushing, Veterinary dentistry

## Abstract

**Background:**

Periodontitis is a common disease in dogs, and daily dental home care in the form of tooth brushing is essential for prevention and treatment. Despite this, many studies reveal low adherence to tooth brushing advice. This study aimed to assess compliance with dental home care among dogs with periodontitis and understand the factors influencing brushing routines. A questionnaire survey was emailed to 63 dog owners whose dogs had been diagnosed with periodontitis, received dental cleaning at the University Animal Hospital, Swedish University of Agricultural Sciences and were given tooth brushing instructions. The survey was supplemented by telephone interviews, resulting in a 57% response rate.

**Results:**

The study presents dog owners' routines, experiences, attitudes, and motivation regarding tooth brushing. Approximately 42% brushed their dogs' teeth daily while others did so less frequently or not at all. Reported challenges, such as uncooperative dogs and difficulty establishing a routine, may explain infrequent brushing.

**Conclusions:**

While the study suggests that owners of dogs with periodontitis tend to follow dental care recommendations better than the general dog owner population, it also reveals that preventive care is inadequate for more than half of the dogs. Given the high prevalence of periodontitis, there's a need for resources to address infrequent tooth brushing. Understanding dog owners' needs can help establish daily brushing as a routine, improving canine oral health and overall well-being.

## Background

Periodontal disease, encompassing both gingivitis and periodontitis, is one of the most common diseases in the dog, with a prevalence rate exceeding 80% in dogs aged three years and older [1–4]. Periodontitis represents the advanced and severe stage of the disease, characterized by the progressive loss of clinical attachment, which, when left untreated, eventually leads to tooth loss [5]. Numerous factors contribute to the development and progression of periodontitis in dogs, encompassing genetic predisposition, age and overall oral hygiene practices [6]. Effective prevention and management strategies encompass regular dental care regimens, including daily mechanical plaque removal through tooth brushing as well as regular professional dental scaling and polishing by veterinary professionals [7]. Given that dental plaque is considered the initiating factor in periodontitis, removal is crucial for the success of prophylactic dental care. Consequently, daily tooth brushing stands as the gold standard for preventing and treating existing periodontal disease [7].

Despite this, several studies indicate that adherence to advice regarding tooth brushing is low [8–10]. This discrepancy between established best practices and actual implementation highlights a significant opportunity for improvement. Addressing this disparity could potentially enhance the quality of life for a substantial portion of the dog population.

The primary objective of this study was to follow up adherence to dental home care among dogs diagnosed with and treated for periodontitis. Additionally, we sought to investigate the various factors that might influence the success or failure of establishing and maintaining a satisfactory tooth brushing routine.

## Methods

A questionnaire survey was constructed according to survey methodology guidelines [11] and distributed via the web platform Netigate (www.netigate.net). Questionnaire items were based on a previously conducted, validated, nation-wide survey by the same research group, enabling comparisons [8, 11]. The questions aimed to assess dental home care and dental health routines after periodontitis treatment.

Participants were selected based on three criteria: a periodontitis diagnosis, treatment at the University Animal Hospital, Swedish University of Agricultural Sciences and receipt of tooth brushing instructions. Written consent for research and educational use of journal entries was obtained from pet owners visiting the university.

The sample group was identified from the University Veterinary Hospital's record systems during 2017–2022 (ProVet Cloud and Trofast). Contact information for 63 dog owners and their dogs' background data was recorded.

The survey was distributed via email, strategically timed for a higher response rate, and remained open from September 27 to October 11, 2022. Respondents could answer only once through various devices. A reminder was sent out by e-mail to non-responders after 5 and 10 days. In cases where the e-mail address was invalid (n = 3) or missing (n = 16), mobile phone numbers were used. On day 6, an SMS reminder was sent out to email recipients (n = 33) who had not yet completed the survey. Additional reminders were sent to all non-responders via SMS after 3 and 7 days.

The number of questions varied from 9 to 14 depending on the response options chosen by the respondent. Most questions were closed with pre-formulated response options. Follow-up open-ended questions were presented based on respondents' previous selections to encourage detailed answers.

For those who did not complete the online survey (n = 35), telephone interviews were offered and conducted with dog owners who agreed and had valid phone numbers, yielding additional responses. Questionnaire items were read out verbatim. These interview responses were added to the overall dataset in the software (Microsoft Excel 2016).

## Results

Out of the 63 dog owners invited to participate in the survey study, responses were received from 32 respondents, with 26 successfully completing the entire survey. Additionally, semi-structured telephone interviews were conducted with 10 dog owners. This combination resulted in a total response rate of 57%, with a total of 36 respondents. Of the remaining 6 respondents who did not complete the survey, 3 answered only the first question and were subsequently excluded. The other 3 respondents answered 1–5 questions each, and their responses have been included in the results.

The dogs' ages ranged from 4 to 15 years, with a median age of 11 years. Their weights varied from 1.1 to 28 kg, with a median weight of 6.7 kg. The time elapsed since the dental procedure at the university animal hospital ranged from 4 to 58 months, with a median duration of 26 months.

### Routines

**Fig. 1:**
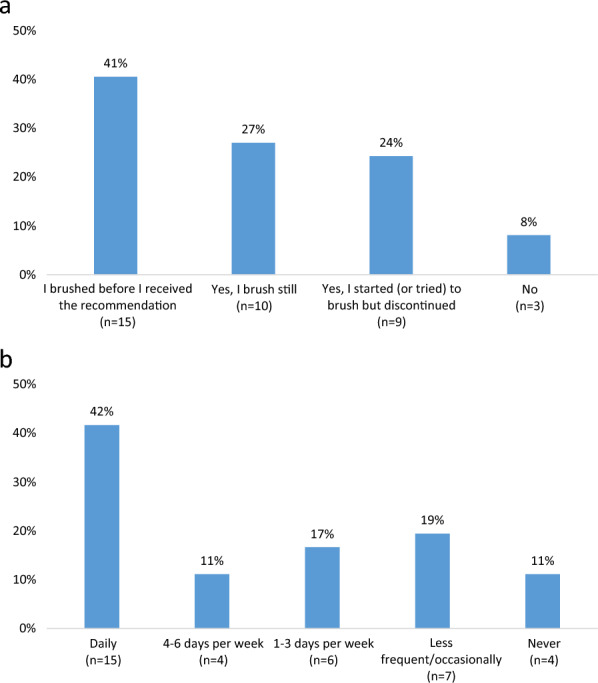
**a** Responses to the question: “In connection with your visit to the dental department, you received home care advice with the recommendation to brush your dog's teeth daily: Did it make you start brushing your dog's teeth?” Note that the question did not include the frequency of brushing. **b** Responses to the question: “How often in the last month have you brushed your dog's teeth with a toothbrush?”

In the study, 15 dog owners (41%) reported that they had already been brushing their dogs' teeth before receiving the recommendation for daily tooth brushing in connection with the dental procedure (Fig. 1a). Following the recommendation, 19 dog owners (51%) initiated tooth brushing. Among these, ten continued to brush, while nine stopped (Fig. 1a).

Among the individuals who were already brushing before the recommendation (n = 15), six respondents mentioned in free text/ interview that they were brushing daily both before and after the recommendation. Three dog owners stated that they previously brushed several times per week but increased to once a day since receiving the recommendation. The remaining respondents brushed three times a week or less. For those who started and continued to brush after the recommendation (n = 10), five reported brushing daily, three almost every day, and two once a week.

Among those who brushed daily or almost daily, half indicated in free text/interview that their primary motivation was the well-being of their dogs and preventing further dental problems. For dog owners who did not start brushing or attempted but then stopped, cited reasons included finding it ineffective, difficult, and a lack of knowledge regarding alternative strategies for successful implementation. In the last month, 15 dog owners (42%) reported daily brushing, four (11%) brushed 4–6 days per week, and six (17%) brushed 1–3 times per week, while the remainder brushed less frequently or not at all (Fig. 1b).

**Fig. 2:**
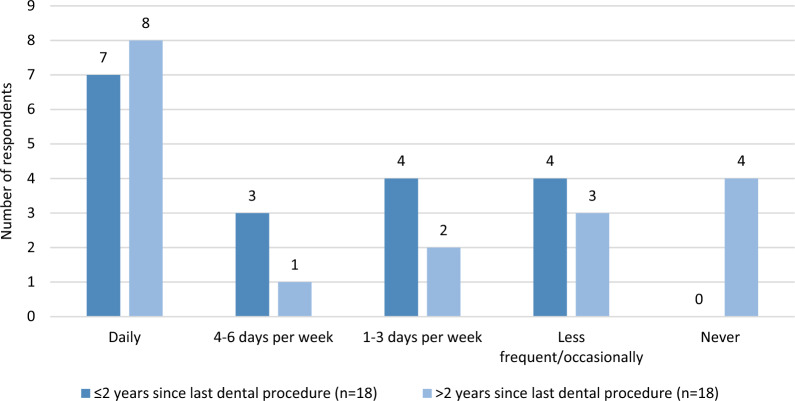
Distribution of reported tooth brushing frequency among 36 dog owner-respondents. This group includes 18 dogs that had their latest dental procedure at the University Veterinary Hospital within the last two years and 18 dogs with procedures performed over two years ago

The tooth brushing frequency comparison between dogs who underwent a dental procedure within the last two years, and those who did so more than two years ago is presented in Fig. 2.

Among the respondents who brushed their dogs' teeth 4–6 days per week or daily (n = 19), their likelihood of continuing at the same level was self-assessed. Fourteen owners (74%) reported the highest likelihood, rating it 10 out of 10, and the mean score for all respondents was 9.3 on a 0–10 scale.

Self-reported factors contributing to this high likelihood included a desire to promote their dog's health, prevent future problems, acknowledge the effectiveness of tooth brushing, consider it a necessity, already established a routine, avoid tooth extractions, reduce the need for additional veterinary visits and anesthesia, and minimize costs associated with dental care.

### Dog owner’s perceptions

The majority (71%) of surveyed dog owners (n = 39) expressed that they perceived their dogs' dental health as very poor (15%) or fairly poor (38%). A smaller percentage (33%) considered it neither good nor bad, while 8% viewed it as fairly good and 5% as very good.

**Fig. 3:**
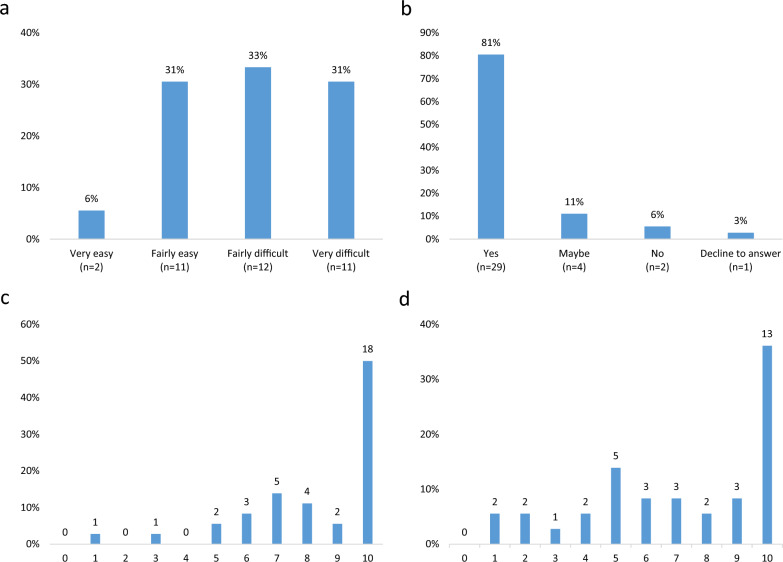
**a** Responses to the question: “How easy or difficult do you find it to be to brush all your dog's teeth?” **b** Responses to the question: “Would you consider brushing your dog's teeth daily?” **c** Responses to the question: “How important is it to you to brush your dog's teeth on a scale from 0 to 10, where 0 means not at all important and 10 means maximally important?” (median = 9.5). **d** Responses to the question: “How sure are you of your ability to brush the dog's teeth if you decide to, on a scale from 0 to 10, where 0 means not at all sure and 10 means completely sure?” (median = 7.5)

In terms of the difficulty experienced in brushing their dog's teeth, 64% reported some level of difficulty, with nearly one-third (31%) finding it very difficult (Fig. 3a).

In the free text/interview responses, 21 dog owners attributed difficulties to having an uncooperative dog, while 9 respondents also mentioned challenges related to accessing certain teeth, particularly the inside of teeth, and molars.

### Attitude and motivation

When asked about the importance of good dental health for their dogs, all respondents indicated it was either very important (84%) or fairly important (16%). The study also revealed that 29 respondents (81%) would consider brushing their dog's teeth daily (Fig. 3b).

The majority also considered tooth brushing important (Fig. 3c).

In free text/interview responses regarding the most important reasons for brushing their dog's teeth, dog owners (n = 36) cited the following: promoting good health (47%), including the dog's general health; preventing the risk of future problems or illness (33%); preventing pain (19%); enabling their dog to eat (17%); and retaining teeth (14%). Note that respondents could mention multiple reasons, resulting in percentages summing over 100%.

Respondents' confidence in their ability to brush their dog's teeth, rated on a scale from 0 to 10, is shown in Fig. 3d. Thirteen dog owners (36%) reported maximum confidence, while others reported varying degrees of certainty.

Among the respondents who scored 0–9 in confidence (n = 23), free text/interview responses indicated a need for more knowledge and advice on handling uncooperative dogs during tooth brushing to prevent scaring, stressing, or injuring the dog. Owners also expressed a desire for tips and tricks to facilitate tooth brushing. Other needs included follow-up at the clinic to assess brushing technique and results, toothbrushes better suited for small dogs, increased knowledge and understanding of the dog's behavior in different situations, and observing improvements, such as reduced tartar buildup.

## Discussion

In this study, it is encouraging to observe that owners of dogs diagnosed with periodontitis for more than four months demonstrated a considerably higher commitment to dental care compared to the general population. However, only 15 of 36 individuals reported brushing their dogs' teeth daily, a practice considered essential for halting the progression of periodontitis, indicating room for improvement.

The predominance of smaller-sized and older dogs in our study aligns with existing research highlighting their overrepresentation in periodontal disease cases [6]. Thus, our study's sample population remains consistent with previous investigations into periodontitis.

### Brushing

After professional dental cleaning and treatment of periodontitis, including subgingival cleaning, initiating daily tooth brushing is crucial to stop disease progression [12, 13]. Every effort should be made to motivate, engage, and support the dog owner in practicing this. In this study, recommendations for daily dental home care, coupled with the dental procedure, initiated tooth brushing for many respondents (51%). Yet, sustaining this practice remains a challenge, with a significant portion (about half) discontinuing after initial attempts. This is in line with results from previous surveys, which showed that 22% [9] and about 26% [8] of the respondents started brushing but then stopped. A large proportion, 41%, stated that they brushed at least to some extent before the recommendation, which is similar to previous results in Sweden where half of the dog owner population reported brushing their dog’s teeth to some extent [8]. Nevertheless, existing daily brushing was somewhat more prevalent in the current study, suggesting a potential pre-existing awareness linked to the dogs’ dental issues.

In this survey, tooth brushing 4–7 days a week was performed in 53% of dogs. This result also agrees with a previous study where 27 of 51 dog owners brushed several times a week after an average of one year, interestingly highlighting that no apparent improvement in tooth brushing adherence has been seen in the last three decades [9]. To the authors’ knowledge, this much earlier study is the only previously published study investigating tooth brushing routines after periodontal treatment in dogs.

Forty-two percent of the respondents in this study brush daily, and 11% state that they brush 4–6 times a week. These proportions are considerably larger compared to the 3.7% of dog owners in the total population who brush their dog's teeth every day and 4.5% who brush 4–6 times a week [8]. This likely reflects dog owners’ heightened motivation driven by a periodontitis diagnosis.

In the free text/interview answer about reasons to brush, the most common comments were the benefit for general health, dental health, pain, and retaining the teeth. This is in line with the general population, where retaining the teeth and the benefit for general health were the most common reasons to brush [8], which should be considered to improve adherence to dental care advice.

### Attitudes

Most people in this study (84%) state that they consider good dental health to be very important, which also seems to be true of dog owners in general (80%) [14]. However, the attitude towards tooth brushing differs between populations, with barely a third of the total dog owner population considering tooth brushing to be very important [8] compared with at least two-thirds in the present study (rated 8–10 on a 10-grade scale). This suggests that owners of dogs with periodontitis are more aware of the importance of tooth brushing for good dental health.

The questionnaire study was supplemented with telephone interviews where, despite conveying the questions and response options verbatim, considerably longer and more comprehensive answers were generated in some cases. The vast majority considered the information given by the veterinarian in the form of home advice to be satisfactory. Motivation to perform dental home care is generally high. Qualitative insights gained from telephone interviews underscore the need for more practical guidance on tooth brushing techniques, especially when dealing with dogs exhibiting fear or stress. Addressing this challenge is essential to enhance adherence to dental care recommendations. That the dog is uncooperative was also the most common reason for difficulties with dental examination in the population at large [14]. This study thus highlights this insecurity among dog owners regarding their ability to perform dental home care (Fig. 3d), which may lead to an increased risk of non-adherence [15].

This study found that four out of five respondents would consider brushing their dog's teeth daily, but only four out of ten say they do. Furthermore, almost one in three owners brush 1–6 days a week. We hypothesize that the threshold to daily brushing is lower among those who already brush to some extent, and targeted interventions may have the largest effect in this group. Such an improvement could potentially have a large positive impact on dogs' dental health and thus also their quality of life [16].

The survey was answered by many respondents a relatively long time after the last dental procedure, but the reported adherence was, despite this, relatively high (Fig. 1b). The vast majority estimated the likelihood that they will continue to brush the dog's teeth at the same level as today as very high (mean > 9 out of a maximum of 10). This suggests that once a tooth-brushing routine is established, it tends to remain stable. The most common free text/interview motivations for this were to avoid future problems, the knowledge that it helps, and that tooth brushing is now a routine. If a good routine is introduced where cooperation works well between the dog and owner, this study shows that it can also be maintained.

### Adherence

Adherence to medical advice is multifaceted, and different explanatory models have been proposed [15, 17–19]. Behavior can be seen as the result of interactions between capability, motivation, and opportunity, where the behaviors generated also influence these three factors [15]. Capability is defined as the individual's mental or physical competence to perform a certain task but also presupposes that he has the knowledge and skill that the task requires. Occasions are all the factors that exist around the individual, which enable or encourage the behavior. Motivation directs behavior but also includes and is influenced by the habits and feelings of the individual [15]. In a given person, it may be enough to change one of these factors to achieve a positive change in behavior.

Dog owners today are generally well informed; therefore, factors other than information alone need to be addressed to increase adherence to dental home care. Client-centered communication, such as motivational interviewing (MI), has been suggested as a way to further increase motivation [18]. The length of time that has passed since the last tooth brushing recommendation may also have an impact on motivation and implementation of dental home care. Regular veterinary visits, with follow-ups where the opportunity is given to correct any misunderstandings, could possibly lead to more successful treatment. With this in mind, we suggest an individualized dental home care plan, depending on what factor(s) are needed for successful implementation for daily tooth brushing.

According to this survey study, the most common reason for inadequate dental home care routines is insufficient cooperation between the dog and the owner, which also agrees with previous findings by the same research group where 79% of Swedish dog owners reported that the dog is unwilling towards dental inspection, leading to difficulties [14]. Additionally, dog owners' self-perceived difficulties with tooth brushing were seen in a thematic analysis as a hindrance to good dental health, and a need for increased knowledge and support was also suggested [20]. While the quantity of brushing in dogs has received some attention, the aspect of brushing quality has not been explored. Moreover, achieving effective tooth brushing in small breeds like Yorkshire terriers has been shown to pose a challenge, even for professional dog caregivers [21]. This underscores the importance of proper tooth brushing techniques, highlighting the need for practical teaching in this regard.

The challenge of insufficient cooperation between dogs and their owners remains a significant obstacle to effective dental home care. As a solution, we propose the delegation of training related to the non-medical aspects of tooth brushing to entities beyond veterinary clinics. Existing dog owner courses, including puppy training classes, can incorporate stress-free oral cavity management and gradual toothbrush introduction. Normalizing dental care as an integral part of responsible dog ownership from an early age may mitigate apprehension surrounding dental care.

### Limitations and strengths

A key strength of this study is that all respondents received uniform care, information, and treatment for periodontitis at the same clinic, reducing potential bias from different settings or consultants. However, as all respondents received similar care at one clinic, results may not fully reflect adherence rates in other veterinary settings.

All responses in our survey were self-reported, which may introduce social desirability bias and potentially overestimate adherence. Additionally, our sample size was relatively small, limiting the generalizability of our findings. Future studies are warranted to delve deeper into various factors that correlate with heightened motivation and improved performance of dental home care in dogs.

## Conclusions

This study showed that owners of dogs with periodontitis seem to follow the recommendation regarding home dental care better compared to dog owners in general. However, due to infrequent brushing among more than half of the dogs, and the high prevalence of periodontitis in dogs, there remains a substantial demand for tools and resources to address the issue of infrequent tooth brushing, for both dog owners and animal care professionals.

Dog owners express a need to learn to manage the dog's resistance to tooth brushing. More knowledge is therefore needed about how this need is to be met. A greater understanding of dog owners' conditions could possibly contribute to getting closer to a solution where tooth brushing becomes a natural part of daily care.

Our findings provide a foundation for future efforts to enhance periodontal disease management and prevention in dogs, ultimately improving the oral health and overall well-being of our canine companions.

## Data Availability

The datasets used and/or analyzed during the current study are available from the corresponding author on reasonable request.

## Declarations

### Prior publication

Data included in this article have previously been published in a Swedish master's thesis for a veterinary degree at the Swedish University of Agricultural Sciences. https://stud.epsilon.slu.se/18863/
